# Structural Characterization and *In Vitro* Antioxidant Activity of Kojic Dipalmitate Loaded W/O/W Multiple Emulsions Intended for Skin Disorders

**DOI:** 10.1155/2015/304591

**Published:** 2015-02-15

**Authors:** Maíra Lima Gonçalez, Diana Gleide Marcussi, Giovana Maria Fioramonti Calixto, Marcos Antonio Corrêa, Marlus Chorilli

**Affiliations:** Department of Drugs and Medicines, School of Pharmaceutical Sciences, UNESP, Rodovia Araraquara-Jaú, Km 1, Campus, 14801-902 Araraquara, SP, Brazil

## Abstract

Multiple emulsions (MEs) are intensively being studied for drug delivery due to their ability to load and increase the bioavailability of active lipophilic antioxidant, such as kojic dipalmitate (KDP). The aim of this study was to structurally characterize developed MEs by determining the average droplet size (Dnm) and zeta potential (ZP), performing macroscopic and microscopic analysis and analyzing their rheological behavior and *in vitro* bioadhesion. Furthermore, the *in vitro* safety profile and antioxidant activity of KDP-loaded MEs were evaluated. The developed MEs showed a Dnm of approximately 1 micrometer and a ZP of −13 mV, and no change was observed in Dnm or ZP of the system with the addition of KDP. KDP-unloaded MEs exhibited ‘‘shear thinning” flow behavior whereas KDP-loaded MEs exhibited Newtonian behavior, which are both characteristic of antithixotropic materials. MEs have bioadhesion properties that were not influenced by the incorporation of KDP. The results showed that the incorporation of KDP into MEs improved the safety profile of the drug. The *in vitro* antioxidant activity assay suggested that MEs presented a higher capacity for maintaining the antioxidant activity of KDP. ME-based systems may be a promising platform for the topical application of KDP in the treatment of skin disorders.

## 1. Introduction

Skin disorders such as skin aging and hyperchromia interfere negatively in the social behavior of people. These disorders are mainly caused by reactive oxygen species induced, for example, by prolonged exposure to UV radiation, establishing a condition in which the oxidative attack of biomolecules is increased [1–6].

The current treatments include laser procedures, dermabrasion and microdermabrasion, and chemical peels. Nevertheless, these treatments present several disadvantages such as persistent erythema, hypopigmentation, keloids, and hypertrophic scars [7].

Another common treatment modality is through the use of cosmetics with tretinoin and hydroquinone drugs; however, many studies indicate that tretinoin can cause skin irritation (e.g., erythema, stinging, burning, and dryness) and hydroquinone can have carcinogenic and mutagenic effects [7].

Therefore, developing new formulations to reduce these degenerative processes and increase people's self-esteem is an ongoing goal. To this end, interest has grown in antioxidant drugs that interfere with the generation of free oxygen radicals and the reactions they trigger [8]. Among these antioxidant drugs, kojic acid (KA) has been highlighted because it presents antioxidant activity by chelating iron ions. KA also presents depigmentation activity by chelating the copper ion present in the active site of tyrosinase, which mediates the formation of melanin from the amino acid tyrosine [9–11].

**Figure 1:**
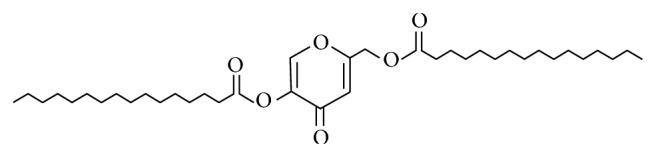
Structural formula of kojic dipalmitate (KDP) [12].

Nevertheless, KA is unstable at high temperatures (40°C) and presents a labile oxidative property against light. Therefore, KA is used as kojic dipalmitate (KDP) (Figure 1), which is hydrolyzed by esterases present in skin cells to promote the* in situ* release of kojic acid [12].

KDP is a liposoluble white powder that is heat- and light-stable over a wide pH range [12].

Due to difficulties in solubilizing fat-soluble drugs such as KDP, a search for strategies to convey the drug is still required. KDP can be incorporated into colloidal carrier systems, such as multiple emulsions (MEs), a single colloidal system consisting of both water-in-oil and oil-in-water emulsion system types. MEs are classified as oil-in-water-in-oil (O/W/O) or water-in-oil-in-water (W/O/W) depending on the dispersed and external phase compositions [13].

Therefore, MEs are being intensively studied as a drug delivery system due to their ability to load lipophilic drugs to increase their bioavailability and protect such drugs against biological degradation and oxidation processes. This can prolong the drug release, which could possibly reduce the required dosages and application time of the formulation [14–16].

Thereby, ME can increase the effectiveness of KDP, acting as a promising drug delivery system to prevent and treat skin disorders [17].

However, MEs contain large and polydisperse droplets that are extremely thermodynamically unstable due to their easy and spontaneous separation into three distinct phases. MEs also tend to undergo coalescence, flocculation, or creaming [18]. One way to obtain stable MEs is to reduce the droplet size through altering the method of preparation and monitoring the system over time to ensure that the droplet size does not increase. Another way to obtain the MEs stable over long periods of time is to choose the correct surfactant system that conserves the droplet size through changes in the interfacial tension between internal aqueous and oil phases in W/O/W emulsions [19].

The aim of this study was to perform the structural characterization of MEs through determining the average droplet size and zeta potential (ZP), performing macroscopic and microscopic analyses, and evaluating the rheological behavior and* in vitro* bioadhesion of the MEs. Furthermore, the* in vitro* safety profile and antioxidant activity of ME-incorporated KDP was evaluated.

## 2. Materials and Methods

### 2.1. Materials

For preparing the formulations, Kojic dipalmitate (KDP) from Via Pharmaceuticals, Brazil, sorbitan monooleate (Span 80) from Bold, Brazil, liquid petrolatum from Teclab, Brazil, sorbitan monooleate ethoxylated 20 OE (Tween 20) from Vetec, Brazil, and water (Milli-Q) were used. In determining the* in vitro* antioxidant activity of the formulations, 2,2-diphenyl-1-picrylhydrazyl (DPPH) purchased from Fluka, Buchs, Switzerland, was employed.

### 2.2. Methods

#### 2.2.1. ME Development

The ME W/O/W system was developed by a two-step process. Initially, the primary W/O emulsion composed of 20% Span 80 (hydrophobic surfactant, HLB (hydrophile-lipophile balance) = 4.3), 45% liquid petrolatum, and 35% water (Milli-Q) was obtained, in which the liquid petrolatum and Span 80 were heated to approximately 40°C and dispersed in water heated to the same temperature. The heat was maintained while the mixture was agitated using magnetic stirrer for 1 minute. The primary emulsion was dispersed into an aqueous solution of Tween 20 (hydrophilic surfactant − HLB = 15) to generate the W/O/W ME composed of 80% of the primary emulsion, 10% of the solution in 40% Tween 20, and 10% water (Milli-Q) [20].

Table 1 shows the components and their concentrations used in the preparation of the KDP-unloaded MEs. To obtain KDP-loaded MEs, 1.25% of the total quantity of liquid petrolatum was reduced from the primary emulsion and an equivalent percentage of KDP was incorporated.

The particle size distribution of a dispersed system depends on the speed of agitation between the dispersed and dispersing phases during the emulsification process and the rate of addition of one phase over another. These parameters were adjusted to obtain a proper system [21].

#### 2.2.2. Physicochemical Characterizations of ME

*(1) Macroscopic and Microscopic Analysis.* The macroscopic appearance, organoleptic properties, and homogeneity of the MEs were evaluated after 24 hours of preparation. The MEs were also observed using an optical Leitz DM RXE microscope (LeicaTM) at 25 ± 0.5°C. The samples were placed on glass slides and covered with a cover slip, and images were captured at 100x magnification.

*( 2) Droplets Distribution and Quantification*

*(A) Optical Microscope.* MEs were placed on a glass slides, covered with a cover slip and observed under a Leitz DM RXE microscope (LeicaTM) coupled to a camera, which allowed for the approximate count of droplets of the MEs samples. Captured images were scanned and analyzed using the Motic Images Plus 2.0 software. 

*(B) Dynamic Light Scattering.* The size and distribution of droplets were determined by an optical particle analyzer (Zetasizer Nano-ZS ZEN3600, Malvern Instruments) through the technique of dynamic light scattering. The samples were diluted in Milli-Q water at a 1 : 20 ratio and placed in the equipment. The analyses were performed in triplicate. 

*(3) Determination of pH.* For the determination of pH, 1 g of formulation was diluted into 10 mL of Milli-Q water. Then, the pH was checked using a digital pH meter (DM-Digimed 23). This assay was performed in triplicate. 

*(4) Determination of Electrical Conductivity.* After the conductivity (Digimed DM-32) was calibrated with KCl 0.1 N, the electrical conductivity was determined by directly inserting the electrode into the samples. This assay was performed in triplicate. 

*(5) Determination of the Zeta Potential.* Zeta potential values were obtained from the derivation of the electrophoretic mobility using the Zetasizer Nano-ZS ZEN3600 optical particle analyzer (Malvern Instruments). The samples were diluted in distilled water at a 1 : 20 ratio and placed in the equipment to be analyzed. The analyses were performed in triplicate. 

*(6) Rheological Study.* Continuous flow was analyzed at 32 ± 0.1°C and in triplicate on a controlled-stress AR2000 rheometer (TA Instruments, New Castle, DE, USA) equipped with parallel plate geometry (40 mm diameter) and a sample gap of 200 *μ*m. Samples of the systems were carefully applied to the lower plate, ensuring that sample shearing was minimized, and allowed to equilibrate for 3 min prior to analysis.

Continual testing was performed using a controlled shear rate ranging from 0.01 to 100 s^−1^ and back. Each stage lasted 120 s, with an interval of 10 s between the curves. The consistency index and flow index were determined using the power law described in (1) for quantitative analysis of flow behavior:

(1) $$\begin{array}{c} \tau =k\cdot \gamma^{\eta }, \end{array}$$

where “*τ*” is shear stress; “*γ*” is shear rate; “*k*” is the consistency index; and “*η*” is the flow index. 

*(7) In Vitro Bioadhesion Test*.* In vitro* bioadhesion of the systems was evaluated using TA.XT Plus texture analyzer (Stable Micro Systems, Surrey, England). A “Hold Until Time” test was used to evaluate the force required for the formulation to leave the animal skin.

The ear skin of domestic pigs was used as an experimental model. The ear skin of domestic pigs purchased from a local slaughterhouse was used as experimental model. The skin was separated from the cartilage with a scalpel and a 500 *μ*m thick layer stratum corneum and epidermis was separated from the adipose tissue with a dermatome (Nouvag TCM 300, Goldach, USA), prior to analysis. Before the test, the skins were maintained in 0.9% NaCl solution for 10 minutes and were affixed to the probe with the aid of rubber rings.

MEs (7.5 g) were placed in Falcon (BD) centrifuge tubes with a capacity of 45 mL and centrifuged at 3500 rpm (Sorval, DuPont Model TC 6) for 3 minutes to eliminate air bubbles and obtain smooth surfaces. The Falcon tube was fixed below the probe. The sample was placed 10 mm below the analytical probe, which was lowered at a constant speed (1 mm/s) until it reached the sample (detected by a 2 mN triggering force) and remained in contact with the formulation for 60 seconds. Then, the probe was removed slowly at a speed of 0.5 mm/s. The force exerted by the probe on the skin surface was determined using the Texture Exponent Lite software to obtain a result curve for the bioadhesive force* versus* time. Assays were performed in triplicate.

#### 2.2.3. *In Vitro* Biological Assays

*(1) Erythrocyte Hemolysis.* The erythrocyte hemolysis assay was performed using the experimental procedure described by Jumaa et al., 1999 [22], and Huang et al., 2008 [23]. Briefly, before use, freshly collected human blood (O positive) was washed three times with a 7.4 pH solution of 0.01 M Tris-HCl containing 0.15 M NaCl (Tris-saline). A suspension containing 1% (v/v) erythrocytes was prepared with packed red blood cells resuspended in Tris-saline. KDP-loaded MEs, KDP-unloaded MEs, and free KDP were dissolved in Tris-saline to a final concentration of 27 *μ*M. As a positive control (100% lysis), a 1% (v/v) Triton X-100 solution was used. After incubation for 1 hour at 37°C, the samples were centrifuged at 3000 ×g for 2 minutes. Aliquots of 100 *μ*L of the supernatant were transferred to 96-well microplates and the absorbance was determined at 405 nm using a BioRad Model 3550-UV (USA) microplate reader. The assay was performed in triplicate. The percentage of hemolysis was calculated using the following equation: % hemolysis = (absorbance of the test sample/absorbance at 100% lysis) × 100.

*(2) In Vitro Nonspecific Cytotoxicity*.* In vitro* testing was performed using J-774 mouse macrophages as a template to analyze the safety profile of the formulations. Cells were seeded in the bottoms of 96-well microplates (Nunclon) at a density of 2.5–10.0 × 10^5^ cells/well and treated for 48 hours with different doses of the KDP-unloaded MEs, KDP-loaded MEs, and free KDP of 1, 5, 10, or 18.6 *μ*M. The cells were washed with PBS after removal of the compounds and cell viability was assessed by colorimetry using formazan (MTT).

The method of 3-[4,5-dimethylthiazol-2-yl]-2,5-diphenyltetrazolium bromide (MTT) is a simple, reliable, and reproducible colorimetric method for measuring mitochondrial metabolic reduction of yellow tetrazolium salt to insoluble formazan crystals in aqueous solutions of viable cells. Cells and MTT (0.4 mg/mL) were incubated at 37°C for 3 hours. Subsequently, the supernatant was removed and formazan crystals were dissolved in DMSO (180 mL). The plates were agitated for 10 minutes and the optical density was measured using a multiwall spectrophotometer operating at 560 nm. The concentrations were tested in triplicate using six additional controls (cells in medium). Cell viability was calculated using the following equation: Cell viability (%) = [OD_560_ (sample)/OD_560_ (control)] × 100.

#### 2.2.4. *In Vitro* Antioxidant Activity

Free radical scavenging activity was evaluated using the 2,2-diphenyl-1-picrylhydrazyl (DPPH) test with modifications [24]. One hundred microliters of free KDP, ME formulations, or control (ethanol; 10–60 *μ*g/mL) was added to 3.9 mL of DPPH solution in ethanol (60 *μ*M). After 30 minutes of storage in a dark place, the absorbance was measured using a spectrophotometer at 517 nm. All measurements were repeated three times. Free radical scavenging activity was calculated using the following formula: % inhibition of DPPH = [(*A*_0_ − *A*_1_)/*A*_0_ × 100], where *A*_0_ = absorbance of control and *A*_1_ = absorbance of sample. The IC_50_ value was determined by plotting the concentration of formulations* versus* the percentage of DPPH that was maintained at a steady state [24].

#### 2.2.5. Statistical Analyses

Data were analyzed using the means and standard deviation and compared by analysis of variance (ANOVA). Tukey's test was used to assess significant differences between samples, where values of *P* < 0.05 were considered statistically significant. The Origin 7.0 SRO program was used for the treatment of the data.

## 3. Results and Discussion

### 3.1. Physicochemical Characterization of ME

**Figure 2:**
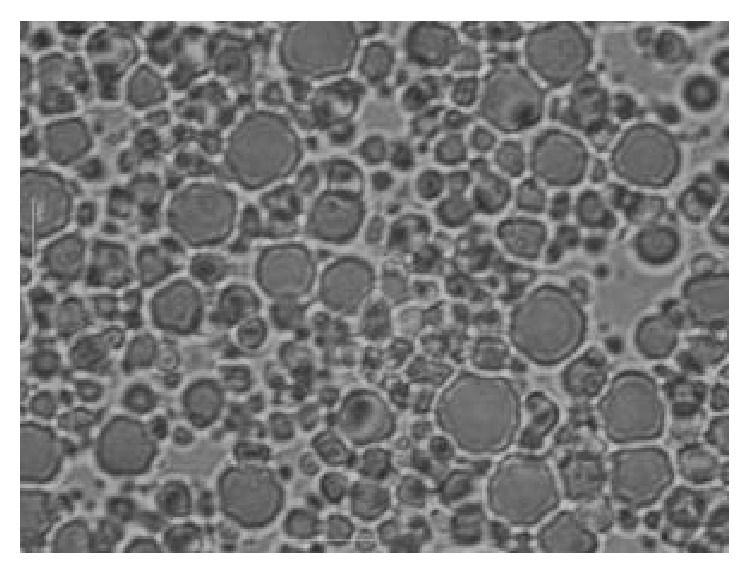
Microscopy of KDP-unloaded MEs (100x) at 25 ± 0.5°C.

The MEs were viscous, white, and opaque with a characteristic odor. By microscopic analysis (Figure 2), larger droplets of less than 10 *μ*m of diameter were observed. At certain times, smaller droplets were observed within the larger droplets, characteristic of ME formulations. Moreover, there was no change in the appearance of the ME formulations with the addition of KDP.

**Figure 3:**
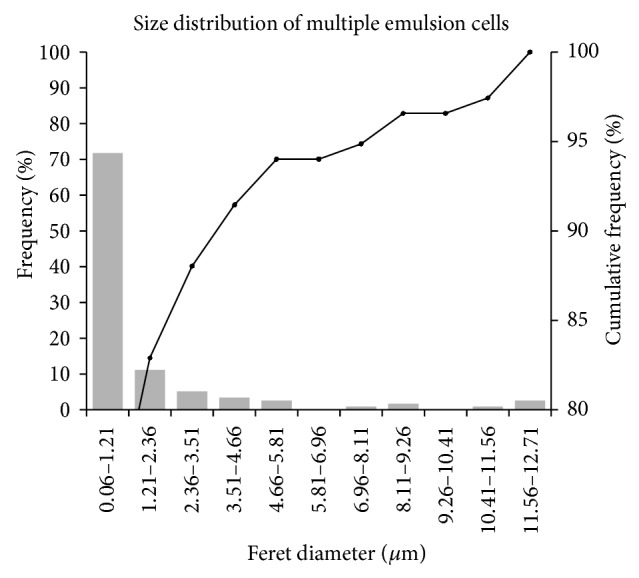
Size distribution of droplets of KDP-unloaded MEs analyzed by optical microscope.

Figure 3 shows the size of droplets and the calculated size distribution of droplets of the KDP-unloaded MEs.

The droplets had a maximum diameter of 12.487 *μ*m and a minimum diameter of 0.056 *μ*m, with an average diameter of 1.534 *μ*m. 72% of the droplets in the formulation were observed to have diameters ranging from 0.06 to 1.21 *μ*m. The addition of KDP did not cause statistically significant changes in droplet size (*P* > 0.05).

Different sizes of droplets can be obtained depending on the method of emulsification selected, explaining the method by which the stability of the system is influenced [25]. Because the droplet size within the emulsion determines the probability that phenomena such as flocculation and coalescence will occur, in general, the smaller the size of the dispersed droplets, the greater the stability of the system [25].

The dynamic light scattering technique showed that KDP-unloaded MEs had two populations of droplets of different sizes. The results showed that 73.3% of the formulation droplets had an average diameter of 1.016 *μ*m and the remaining 23.7% had an average diameter of 350 nm, as could be observed morphologically using an optical microscope.

Through the statistical ANOVA test, the average pH values of the KDP-unloaded MEs (pH = 4.62) and KDP-loaded MEs (pH = 4.55) were found to be equal (*P* > 0.05), leading to the conclusion that the incorporation of KDP induced no significant change in pH. These pH values are well tolerated by the skin, which presents a slightly acid pH (4.5 to 5.5) [20].

The pH is an important parameter to consider because large variations in the pH values of the formulations can cause chemical incompatibilities between the ingredients of the formulation, therefore compromising the efficacy and safety of the active compounds. In addition to being a parameter related to the intrinsic stability of the formulation, it is essential to control the pH of topical application products to ensure that over time they do not cause changes in the microflora of the skin or induce irritation or skin sensitization, which could degrade the product and reduce its activity [21].

Through the statistical ANOVA test, the average electrical conductivity of KDP-unloaded MEs (34.2 *μ*S/cm) and that of KDP-loaded MEs (31.90 *μ*S/cm) were found to be equal (*P* > 0.05), leading to the conclusion that the incorporation of KDP induced no significant change in the electrical conductivity of the ME system.

In terms of conductivity, the electrical properties of oil and water are extremely distinct and can be used to distinguish whether the ME external phase is aqueous or oily. In accordance with the results obtained, electrical conductivity superior to 30 *μ*S/cm, it was confirmed that the external phase is aqueous and that the ME formulation is a W/O/W type of emulsion [26].

The KDP-unloaded MEs showed an average ZP of −13 mV. The addition of KDP did not cause statistically significant changes in the ZP (*P* > 0.05).

The ZP results from the electric charges on the surface of the particle and is related to the physical stability of the colloidal system [27]. At greater absolute values (more than +30 mV or less than −30 mV), the ZP of a system indicates the tendency of the particles to repel each other, which may indicate that the system is stable. If, however, the absolute values for the ZP are low or zero, the particles tend to agglomerate and the system can easily flocculate [28]. In this case, the formulation should be monitored over time to check whether the intermediate ZP values (−13 mV) indicate an unstable system.

Semisolids products can exhibit a wide range of rheological behaviors. A good understanding of formulation rheology is very important to the design, selection, and operation of the equipment involved in evaluating the release performance and administration characteristics of the drug. Furthermore, rheological studies can provide useful information on the stability and microstructure of emulsions. MEs can display flow characteristics depending on the fraction volume, droplet size and distribution, and the viscosity of the dispersed phase [29–32].

**Figure 4:**
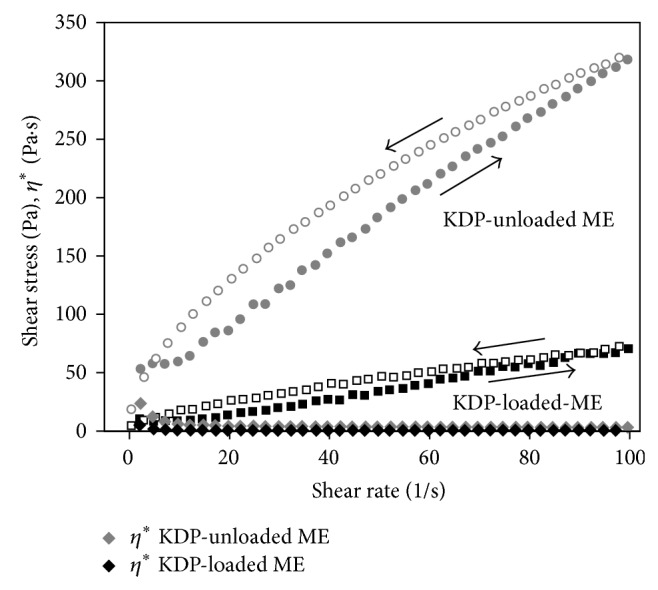
Flow rheograms of KDP-unloaded ME (○) and KDP-loaded ME (□). Closed symbol represents up curve and open symbol represents down curve. *η* ^*^ (◊) is viscosity. Standard deviations have been omitted for clarity; however, in all cases, the coefficient of variation of triplicate analyses was less than 10%. Data were collected at 32 ± 0.25°C.

The relationship between the shear rate (Pa) and shear stress (1/s) of MEs is presented in Figure 4.

From the data obtained using (1) and shown in Table 2, KDP-unloaded MEs were demonstrated to exhibit “shear thinning” flow behavior (*n* < 1), whereas KDP-loaded MEs exhibited Newtonian behavior (*n* = 1).

The KDP-unloaded ME formulation behaved as a pseudoplastic shear thinning fluid because its viscosity (*η* ^*^) decreased with increasing stress. This may be due to the breaking of organized structures that promote the formation of droplets, which are less organized structures [33].

However, the incorporation of the KDP drug had a strong influence on the formulation, leading the KDP-loaded ME formulation to behave as a low-viscosity Newtonian fluid. This phenomenon is related to the intense reduction in the droplet size with the introduction of KDP, which thinned the flow and resulted in a higher diffusion coefficient than when larger oil droplets are present.

Furthermore, the rheogram showed that the up and down curves did not overlap. The up curve was below the down curve, which is typical and characteristic of antithixotropic materials.

Antithixotropic properties occur because the steady application of shear stress increases the apparent viscosity, whereas cessation of the applied stress decreases the viscosity back to its initial value in a time-dependent manner [30].

The increase in the apparent viscosity of the ME formulations with time at a constant shear was most likely due to the reorganization of the droplets by strong forces. The high viscosity of the MEs could retard the free motion of the droplets, thus delaying creaming, flocculation, and coalescence of the system [33]. The conclusion from these results is that stable MEs can be obtained with the components used in this study only under the application of vigorous stirring during preparation so that viscosity is incorporated and the destabilization of the system is avoided.

The KDP-unloaded MEs and KDP-loaded MEs were evaluated for their changes in bioadhesion to determine whether the addition of KDP alters the bioadhesive ability of the ME system.

Table 3 shows the bioadhesion test results of KDP-unloaded MEs and KDP-loaded MEs.

The results demonstrate that the incorporation of KDP did not influence the bioadhesion of the MEs. Moreover, the formulations showed values very close to the bioadhesion of polyacrylic acid bioadhesive hydrogels used in previous studies [32].

Thus, MEs have properties that favor the interaction between its components and the epithelial cells of the skin.

The investigation of the strength of bioadhesive pharmaceutical formulations for topical use is extremely important because bioadhesive formulations remain in contact with the biological substrate for longer periods of time, which may lead to a gradient of drug concentration at the site of action and therefore improve the clinical performance of the treatment.

### 3.2. *In Vitro* Biological Assays

Before exposing users to the product, the potential irritation of a new topical preparation should be investigated to avoid inducing skin irritation. For ethical, legal, and financial reasons,* in vivo* tests were banned for testing cosmetics and formulations. Thus, in recent years, various* in vitro* assays have been conducted to test the products prior to realizing there* in vivo* safety profiles [34].

Erythrocyte-induced hemolysis* in vitro* is considered to be a simple and reliable measure for estimating the membrane damage caused* in vivo* because erythrocytes are easily isolated by centrifugation [35].

**Figure 5:**
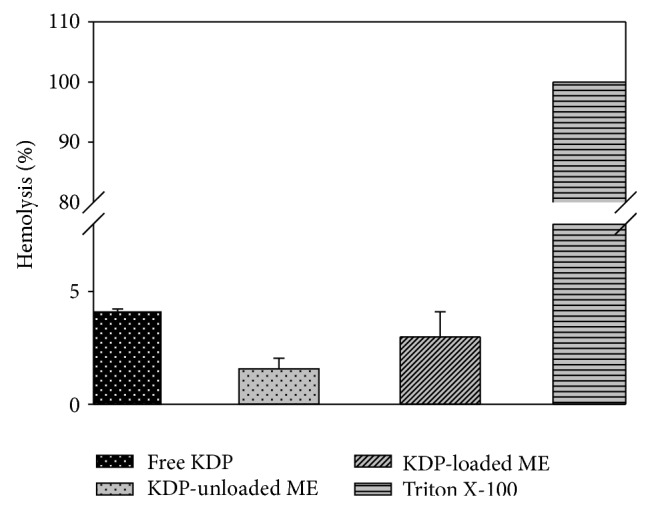
Percentage of hemolysis of red blood cells after treatment with free KDP, KDP-unloaded MEs, and KDP-loaded MEs.

*In vitro* cytotoxicity assays using the hemolysis of red blood cells were used to assess the safety profile of the formulations and they are presented in Figure 5.

Free KDP caused lysis of 4.09 ± 0.13% of the erythrocyte membranes. KDP-unloaded MEs caused lysis of 1.57% ± 0.47% of the erythrocyte membranes. The incorporation of KDP in ME was 2.98 ± 1.12% and showed decreased erythrocyte lysis compared to the free KDP. Thus, all systems showed a tolerable hemolysis of erythrocytes. The positive control was represented by 100% hemolysis of erythrocytes using Triton X-100, a known hemolytic agent. The study results indicate that the treatment developed using the ME system showed low toxicity and may therefore be a potential alternative to current therapeutic applications [23].

**Figure 6:**
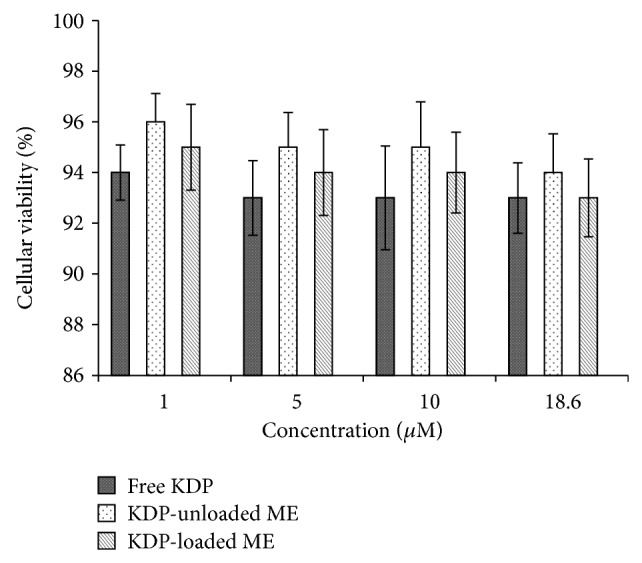
Percentage of cell viability after treatment with free KDP, KDP-unloaded MEs, and KDP-loaded MEs.

*In vitro* cytotoxicity was performed using J-774 mouse macrophages as a cellular model. The data are shown as the percentage of cell viability (Figure 6).

The cell viability assays showed that more than 93% of the normal macrophages treated with free KDP survived. Moreover, neither the KDP-unloaded MEs nor the KDP-loaded MEs showed toxic activity. Therefore, the results indicate the safety and biocompatibility of the formulations and free KDP with eukaryotic cells [36–38].

### 3.3. *In Vitro* Antioxidant Activity

**Figure 7:**
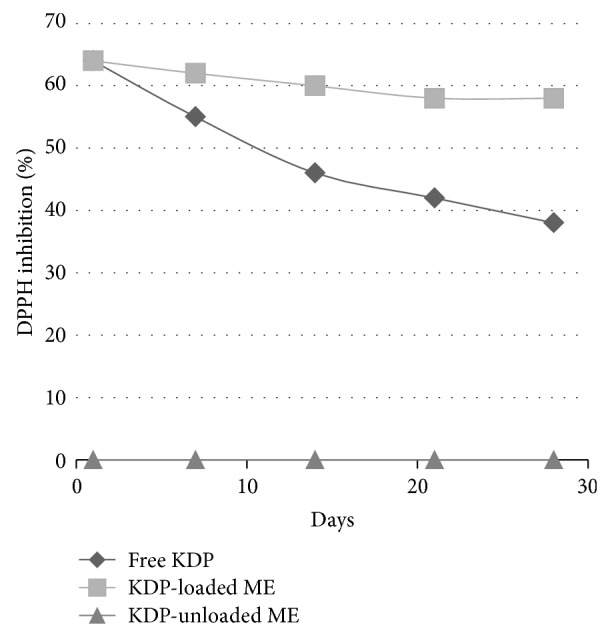
Percent inhibition of the 2,2-diphenyl-1-picrylhydrazyl (DPPH) radical by the formulations over a period of 28 days.

The formulations with or without the addition of KDP were evaluated for their* in vitro* antioxidant activity over a period of 28 days. The results obtained are shown in Figure 7.

The antioxidant activity was measured based on the methodology of Blois, 1958 [24], in which the stable radical DPPH is reduced by antioxidants. The results of this study are expressed as the percent inhibition of DPPH (%).

Over the period of 28 days, there was a decrease in the antioxidant power of all the experimental groups, with the most marked decrease for the free KDP. Differences between the samples were significant (*P* < 0.05) and the lesser destabilization of the samples is most likely due to the increased stabilization of the KDP-loaded ME formulation.

Therefore, the ME formulation maintained the antioxidant properties of KDP, which makes MEs a promising vehicle for the incorporation of KDP to facilitate its topical application in the treatment of skin aging.

## 4. Conclusion

Through physicochemical characterization assays, it was confirmed that the ME system developed was of the W/O/W type with a Dnm of approximately 1 *μ*m and a ZP of −13 mV. No change in Dnm, ZP, or the stability of the system was observed after the addition KDP. KDP-unloaded MEs exhibited “shear thinning” flow behavior, and the incorporation of the KDP drug strongly influenced the formulation, leading the KDP-loaded MEs to behave as a Newtonian fluid. This phenomenon can be related to the intense reduction in the droplet size in the presence of KDP, which thinned the flow. However, both flow types are characteristic of antithixotropic materials. The properties of MEs favor the interaction between the ME system and the epithelial cells of the skin. KDP incorporation did not influence the bioadhesion of the MEs, and KDP-loaded MEs showed low toxicity and a good antioxidant activity. These results indicated that this is a promising system for using KDP in the treatment of skin aging.

## Figures and Tables

**Table 1:** Composition (%) of the KDP-unloaded ME formulations used in this study.

Formulations	Milli-Q water (%)	Span 80 (%)	Liquid petrolatum (%)	Tween 20 40% (%)	Primary emulsion (%)
Primary emulsion	35	20	45	—	—
Multiple emulsion	10	—	—	10	80

**Table 2:** Consistency index (*k*) and flow index (*n*) of KDP-loaded MEs and KDP-unloaded MEs.

Formulations	*k*	*n*
KDP-loaded ME	0.63	1.02
KDP-unloaded ME	9.42	0.76

**Table 3:** Bioadhesion of KDP-unloaded MEs and KDP-loaded MEs.

Formulations	Bioadhesive work (N·s)	Bioadhesive force (N)
KDP-unloaded MEs	0.0166 ± 0.0016	0.0103 ± 0.0029
KDP-loaded MEs	0.0163 ± 0.0025	0.0099 ± 0.0006
